# Prediction of oral squamous cell carcinoma based on machine learning of breath samples: a prospective controlled study

**DOI:** 10.1186/s12903-021-01862-z

**Published:** 2021-10-06

**Authors:** Sophia Mentel, Kathleen Gallo, Oliver Wagendorf, Robert Preissner, Susanne Nahles, Max Heiland, Saskia Preissner

**Affiliations:** 1 Department Oral and Maxillofacial Surgery, Charité – Universitätsmedizin Berlin, Corporate Member of Freie Universität Berlin, Humboldt-Universität zu Berlin, and Berlin Institute of Health, Augustenburger Platz 1, 13353 Berlin, Germany; 2 Science-IT and Institute of Physiology, Charité – Universitätsmedizin Berlin, Corporate Member of Freie Universität Berlin, Humboldt-Universität zu Berlin, and Berlin Institute of Health, Philippstrasse 12, 10115 Berlin, Germany

**Keywords:** Breath analysis, Head and neck cancer, Oral squamous cell carcinoma, Oral cancer, Machine learning, Gas chromatography–ion mass spectrometry, Volatile organic compounds

## Abstract

**Background:**

The aim of this study was to evaluate the possibility of breath testing as a method of cancer detection in patients with oral squamous cell carcinoma (OSCC).

**Methods:**

Breath analysis was performed in 35 OSCC patients prior to surgery. In 22 patients, a subsequent breath test was carried out after surgery. Fifty healthy subjects were evaluated in the control group. Breath sampling was standardized regarding location and patient preparation. All analyses were performed using gas chromatography coupled with ion mobility spectrometry and machine learning.

**Results:**

Differences in imaging as well as in pre- and postoperative findings of OSCC patients and healthy participants were observed. Specific volatile organic compound signatures were found in OSCC patients. Samples from patients and healthy individuals could be correctly assigned using machine learning with an average accuracy of 86–90%.

**Conclusions:**

Breath analysis to determine OSCC in patients is promising, and the identification of patterns and the implementation of machine learning require further assessment and optimization. Larger prospective studies are required to use the full potential of machine learning to identify disease signatures in breath volatiles.

**Supplementary Information:**

The online version contains supplementary material available at 10.1186/s12903-021-01862-z.

## Background

Approximately 354,864 new cases of oral cancer are diagnosed annually, and the number, which was associated with 177,384 deaths in 2018, is steadily increasing [1]. About 90% of oral cancers diagnosed are oral squamous cell carcinomas (OSCCs), which result in malignancies in men at least twice as often as women [2]. In Germany, the 5-year survival rate of patients diagnosed with OSCC varies between 63% (female) and 47% (male) [3]. Mortality is associated with the high recurrence rate and metastases of OSCC, and the delayed diagnosis of the disease [4, 5]. Only one-third of OSCC are discovered at an early stage (0–I) [6, 7]. Therefore, the development of tests that enhance our capacity to screen high-risk (e.g. heavy tobacco and alcohol abuse) [8] and post-therapy patients is of great interest.

Breath analysis is not burdensome to patients, and is a rapid, non-invasive and inexpensive cancer screening tool. Its use has already been determined to be a promising approach to detect and differentiate various diseases, gastrointestinal conditions, and cancer types, such as lung, breast, colorectal cancer [9–12]. In every exhaled human breath, specific volatile organic compounds (VOC) that are byproducts of normal cell metabolism can be identified. These compounds are also present in biofluids such as blood, saliva, urine and feces [13, 14]. The concentrations and types of VOCs present in the exhaled breath of cancer patients compared to healthy individuals may differ based on differences in levels of oxidative stress, which are enhanced in tumor tissues [15, 16]. Gas chromatography coupled with mass spectrometry (GC–MS) is considered the gold standard for VOC screening. However, the E-nose technique, which is based on breath analysis, has produced promising results in OSCC patients as well [17–19]. Schmutzhard et al. [20] showed that a significant difference between VOC data from cancer patients relative to the two control groups could be detected using proton transfer-reaction-mass spectrometry (PTR-MS). A study published by Hakim et al. [21] revealed that data produced via GC–MS could be used to detect statistically significant differences between the breath compositions of three evaluated groups (OSCC/lung cancer/control). Further, the authors were able to distinguish groups using a Nanoscale Artificial Nose. Gruber et al. [22] published a feasibility study comparing OSCC patients, benign tumor patients and healthy controls that identified three potential biomarkers of OSCC using GC–MS. Bouza et al. [23] concluded that aldehyde compounds had the capacity to function as OSCC biomarkers when detected using solid-phase micro extraction followed by GC–MS. Further, Hartwig et al. [24] published a pilot study that revealed the absence of three specific VOCs after curative surgery for OSCC when compared to a patient’s initial GC–MS spectrum, which indicated a correlation between OSCC and the specific VOCs identified.

Machine learning is a computational branch that emulates human intelligence by learning from big data, and is applied in various fields, such as finances, entertainment or biological and medical applications to detect patterns which are hard or impossible to see for the human eye [25]. During the last years, a wide range of machine learning approaches were developed for the early diagnosis of different kinds of cancer from images. These include breast cancer detection by analyzing digitized images of fine needle aspirates of breast masses [26], lung cancer prediction from computed tomography images [27, 28] and brain cancer detection using magnetic resonance imaging [29]. Recent developments even include mobile applications for the detection of skin diseases via user-provided images, which are widely applicable and easy to use [30].

The aim of this study was to evaluate breath samples before and after surgery in a larger cohort using machine learning to compare OSCC patients with healthy smokers to optimize the identification of signatures of OSCC using a recently introduced gas chromatography–ion mobility spectrometry (GC–IMS)-based method. Further, we aim to enhance the applicability of the test by improving the detection of OSCC specific IMS signals that may be used to determine a VOC signature in future studies.

## Patients and methods

### Study population

In this prospective controlled study we collected breath samples from 55 patients with potential OSCC, as well as 50 breath samples from healthy controls. The Ethics committee of the University formally approved the study (EA1/203/19). Written informed consent for study participation was obtained from study participants. All methods were carried out in accordance with relevant guidelines and regulations.

Patients between the age of 18 and 85 with OSCC in the oral cavity and oropharynx with surgical therapy pending were included in the study. Exclusion criteria included a diagnosis of other severe internal accompanying diseases, HIV infection and a Karnofsky performance status scale of less than 50%. All participants in the control group were required to be daily smokers, at least 18 years old and lack known malignant pre-existing conditions.

### Sampling

Standardization of sampling in terms of location and patient instruction was known to be crucial from the literature and our pre-tests. Patients were instructed to fast at least 6 h before sampling and refrain from cleaning their teeth with toothpaste or mouthwash. Samples were also taken in a healthy control group under the same conditions and instructions. Patients were instructed to breathe a few times through the slightly opened mouth. Air from each participant’s breath was collected using a 5 mL Luer syringe directly from the mouth. During transport, each syringe was closed with a stopper to prevent contamination. The procedure was repeated twice. All samples were analyzed within 20 min. Additionally, two syringes filled with room air were analyzed.

If analysis within 20 min was not possible (n = 2), the sample was transferred to a single use mylar bag (QuinTron, Milwaukee, WI, USA), stored at room temperature and analyzed within 24 h [31]. During analysis we made sure that these samples did not differ significantly from the other samples. Sampling always took place the morning before surgery or panendoscopy.

### Gas chromatography/ion mass spectrometry (GC/IMS)

Breath sample analysis was executed using BreathSpec® (GAS Dortmund, Germany). The device facilitated two-fold separation via GC combined with IMS to detect gaseous compounds in a mixture of analytes. VOCs were pre-separated based on their retention times via GC and detected using an IMS electrometer based on specific drift times needed to travel a fixed distance (drift tube) in a defined electric field.

Samples were injected using a 5 mL-Luer-syringe via a Luer-Lock-Adapter into the BreathSpec® (GAS Dortmund, Germany). Samples were heated to 60 °C while passing through the first transfer line and were pumped into the sample loop (40 °C). A carrier gas transported the sample gas in the loop to the GC column (60 °C). During the first separation, different VOCs pass through the GC capillary column (30 m × 0.53 mm, 0.5 μm) at various speeds due to their different retention times. Next, when passing through the second transfer line (60 °C), separated compounds consecutively are fed into the IMS ionization chamber (45 °C). The first separation reduces levels of competition between analytes for reactant ions and enhances the sensitivity of IMS detection. VOCs are softly chemical-ionized initiated by a low-radiation tritium (H3) source. The collision between fast electrons emitted from the β-radiator (H3) with an inserted reagent gas, which is followed by a cascade of reactions, generates reactant ions. This forms the so-called reaction ion peak (RIP), which represents the number of ions available. The chemical ionization of analytes by reactant ions creates specific analyte ions, as long as the affinity of the analyte to the reactant ion is greater than its affinity to water, which is typical for all heteroatom-organic compounds. Specific analyte ions travel at atmospheric pressure versus a flow of inert drift gas in the drift tube, and the resulting ion current is measured using an electrometer (drift length: 98 mm, electrical field strength: 500 V/cm). IMS measurements are extremely fast (30 ms/spectrum). The mass and geometric structure of an ion determines the drift time of each substance. Therefore, IMS can differentiate isomeric molecules.

To perform analyses, two breath samples and two room air samples were taken from each participant. One sample of the patient’s breath and one of the surrounding air was analyzed using the positive drift voltage IMS mode and one of each of the breath and air samples were assessed using the negative drift voltage mode. The total processing time for one sample was 10 min.

### VOC analysis

For visualization and analysis of data, a software provided by the manufacturer was used (VOCal, Dortmund, Germany). GC separation of VOCs divided compounds based on their retention times in the capillary, which resulted in an offset feed into IMS and generated coordinates on the y-axis of the pictorial representation. IMS was used to separate compounds according to their specific drift times in an electric field, which have been displayed as coordinates on the x-axis. These data produce a two-dimensional visualization scheme. The quantification of compounds was performed down to the low parts per billion (ppb) level, and data were used to create a z-axis in the software. Signal intensity was correlated with the analyte concentration of a sample. For analysis, individual signals were marked manually, and signal intensity changes and the presence of recurring patterns were identified using tools in the software.

### Machine learning

To work with the 2-dimensional images, which were produced using the manufacturer’s software, they were first transformed into integer arrays. To achieve this, the “Image module” from the Python library pillow (https://pillow.readthedocs.io/en/stable/reference/Image.html) was used to load the images. After successfully transferring the images into the Python script, they were subsequently converted to numpy arrays (https://numpy.org/), using the function “asarray”. The resulting array consists of integer values specifying the color of each pixel in RGB format (https://htmlcolorcodes.com/), so for each pixel in the original image, three color values are produced that represent its respective amount of red, green and blue. Furthermore, to assure all images were of the same size, all images were reshaped to a standard format of 200 × 200 pixels using the numpy function “resize”, since even a difference in size by one pixel could potentially influence the results. As a last step, to ensure an equal importance of each feature, the multidimensional array representing the color values was collapsed into a 1-dimensional array using the numpy function “ravel”.

To identify the best performing classifier, a number of different models were evaluated, including random forest [32], logistic regression [33], K nearest neighbors [34], and linear discriminant analysis [35]. All methods are implemented in the Python library Scikit-Learn (https://scikit-learn.org/stable/), and used with the respective recommended initial parameters. To build each model, depending on the comparison in question, the images were separated into the two categories of “true” and “false” respectively. To train and evaluate the performance of each model, the data was split into training and test set. The training set was used as input for the machine learning model, while the test set was hold back so it remained completely unknown to the machine learning model. After finishing the training, each image in the test set was then predicted by the machine learning model to be “true” or “false” and it was assessed if the model did the correct prediction. The prediction accuracy of each model was analyzed multiple times with varying sizes of training and test set. Initially, a tenfold cross-validation was performed [36], where the data set is split into 10 equally sized parts. Each of the 10 subsets is then used once as test set, with the remaining 9 parts being the training set for this specific case.

Additionally, the recall was evaluated using the leave-one-out methodology [37]. In this case, the test set consists only of a single data sample, while all remaining samples were used as a training set to build the model. Here, each image was used once as a test sample and therefore left out while training the model. Subsequently, the left-out test sample was predicted and the prediction was determined to be either true or false.

## Results

### Manual VOC evaluation

**Fig. 1:**
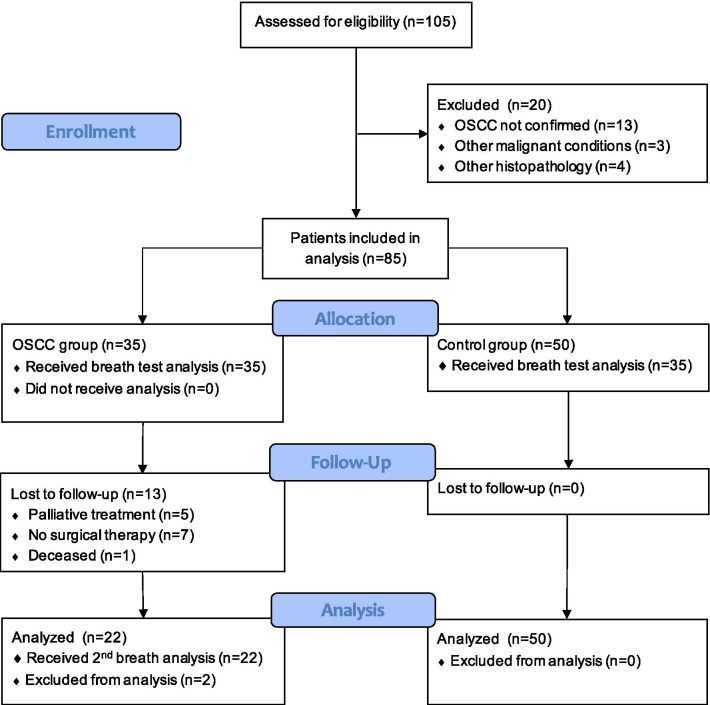
CONSORT flow diagram

The study population consisted of 55 patients with suspected OSCC before surgery and 50 healthy control subjects. After applying exclusion criteria, some patients could not be included in the final data analysis (Fig. 1).

The preoperative analysis consisted of 35 patients (24 men and 11 women) with an average age of 67.2 years. According to their medical history, 21 participants were smokers (60%), four were former smokers, ten were non-smokers (28.6%) (Tables 1 and 2).

**Table 1:** Characteristics of OSCC patients: Age, sex (m: male, f: female), smoking habits (+: smoker, −: non-smoker, +a: former smoker), ICD 10 code (*: recurrence), TNM classification

Patient no	Age	Sex	Smoker	ICD 10	Location	TNM
1	76	M	−	C03.1	Lower gum	T4a N0 M0
2	52	M	+	C03.1	Lower gum	T4a N1 M0
3	61	M	+	C02.1	Border of tongue	T2 N0 M0
4	59	M	+	C04.0	Anterior floor of mouth	T1 N0 M0
5	62	M	+	C04.1	Lateral floor of mouth	T3 N3b M0
6	53	M	+a	C02.1	Border of tongue	T1 N0 M0
7	80	F	+	C03.0	Upper gum	T4a N2c M0
8	60	F	−	C02.1	Border of tongue	T1 N0 M0
9	76	F	+	C04.0	Anterior floor of mouth	T1 N0
10	86	F	+a	C02.1	Border of tongue	T3 N3b M1
11	74	F	+	C04.0	Anterior floor of mouth	T1 N0 M0
12	89	M	+	C05.0*	Hard palate	T4a N0 M0
13	81	M	−	C03.0	Upper gum	T4a N0 M0
14	63	M	+	C02.1	Border of tongue	T3 N3b M0
15	76	M	+	C04.8	Overlapping lesion of floor of mouth	T3 N1 M0
16	61	M	+a	C03.1	Lower gum	T4a N0 M0
17	63	F	+	C14.8	Overlapping lip, oral cavity	T4a N1 M0
18	75	M	−	C05.1	Soft palate	T1 N1
19	71	M	−	C06.0	Cheek mucosa	T1 N0 M0
20	72	M	+	C03.1	Lower gum	T4a N3b M0
21	49	M	+	C03.1	Lower gum	T4a N0 M0
22	58	F	−	C02.1	Border of tongue	T1 N0 M0
23	62	M	+	C06.0	Cheek mucosa	T4 N2 M0
24	83	M	+	C02.0	Dorsal surface of tongue	T2 N0 M0
25	63	F	+	C04.8	Overlapping lesion of floor of mouth	T4b N2 M0
26	63	F	+	C05.0	Hard palate	T3 N2c
27	88	M	+a	C03.1	Lower gum	T4b N2 M0
28	43	M	+	C02.0*	Dorsal surface of tongue	T4 N0 M0
29	81	F	−	C06.0	Cheek mucosa	T4a N0 M0
30	44	M	+	C05.0	Hard palate	T4a N2 M0
31	83	M	+	C04.9	Floor of mouth	T2 N0 M0
32	58	M	−	C04.8*	Overlapping lesion of floor of mouth	T3 N0 M1
33	62	M	−	C02.1	Border of tongue	T1 N0 M0
34	65	M	+	C04.0	Anterior floor of mouth	T2 N0 M0
35	61	F	−	C03.1	Lower gum	T1 N0 M0

**Table 2:** Summary table of the cohort

	Male	Female	Smoker	Non-smoker	Former smoker	T1/2	T3/4
Before surgery	24	11	21	10	4	14	21
After surgery	15	7	13	6	3	9	13

Postoperative sampling was carried out in 22 patients (some were lost during follow-up, Fig. 1). Breath samples were taken approximately 12 days after surgery. The control group included 50 healthy smokers (25 men and 25 women), with an average age of 55 years.

**Fig. 2:**
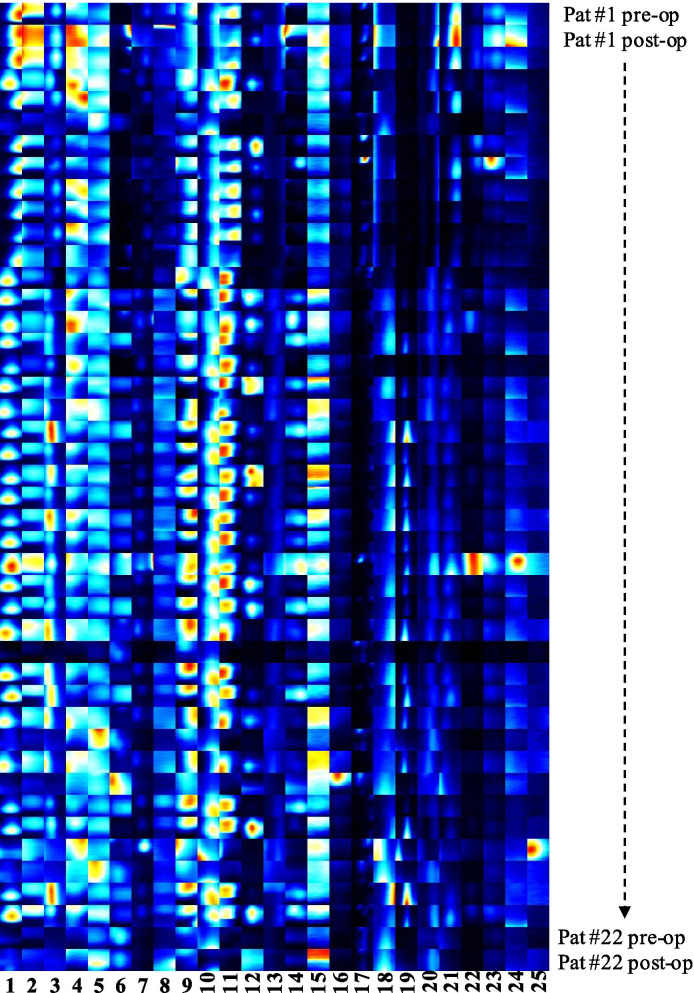
Comparison of pre- and postoperative measurements. The heat map shows 25 areas of interest revealed using 44 measurements of 22 patients before and after surgery in negative drift mode. Certain VOCs are present in all samples (areas 1, 2, 5, 9), and display different signal intensity (concentration), others are inconsistently observed (e.g. areas 6, 12, 14, 17, 20), and some VOCs are present in exclusively pre- or postoperative samples (e.g. areas 24 and 25)

To compare the occurrence of different VOC areas in the population and between preoperative and postoperative patients, signals in the visual representation that corresponded to substances in analyzed air were manually marked in all measurements. Data from one patient were placed in chronological order and compared using marked signal areas. It was revealed that certain substances occurred in almost all patients (areas 1, 2, 5, 9), while others appeared regularly, but not in every sample. Others were found very sporadically (areas 6, 12, 14, 17, 20) and some areas were exclusively present in pre- or postoperative samples (areas 24, 25). (Fig. 2, Table 3). Some areas were overlapped by VOCs of disinfectants (e.g. ethanol) and were therefore excluded from further analysis.

**Table 3:** Intensity changes in IMS signals between pre- and postoperative measurements: Area no. 1–25 (see Fig. 2), preoperative and postoperative signal count (n of 22 patients with IMS signal in measurement), Δ of IMS signal intensity (0: no or little changes, +: increased signal, −: decreased signal)

Area no	Preoperative signal count	Postoperative signal count	Δ of IMS signal
1	21/22	20/22	0
2	21/22	19/22	0
3	20/22	17/22	+
4	21/22	17/22	−
5	20/22	18/22	+
6	13/22	19/22	+
7	20/22	14/22	0
8	16/22	13/22	0
9	22/22	22/22	0
10	16/22	13/22	0
11	20/22	15/22	−
12	12/22	15/22	+
13	15/22	12/22	−
14	13/22	13/22	0
15	21/22	16/22	−
16	20/22	11/22	−
17	7/22	11/22	0
18	11/22	14/22	+
19	10/22	12/22	+
20	4/22	13/22	+
21	17/22	17/22	0
22	9/22	9/22	0
23	19/22	18/22	0
24	0/22	2/22	0
25	1/22	0/22	0

**Fig. 3:**
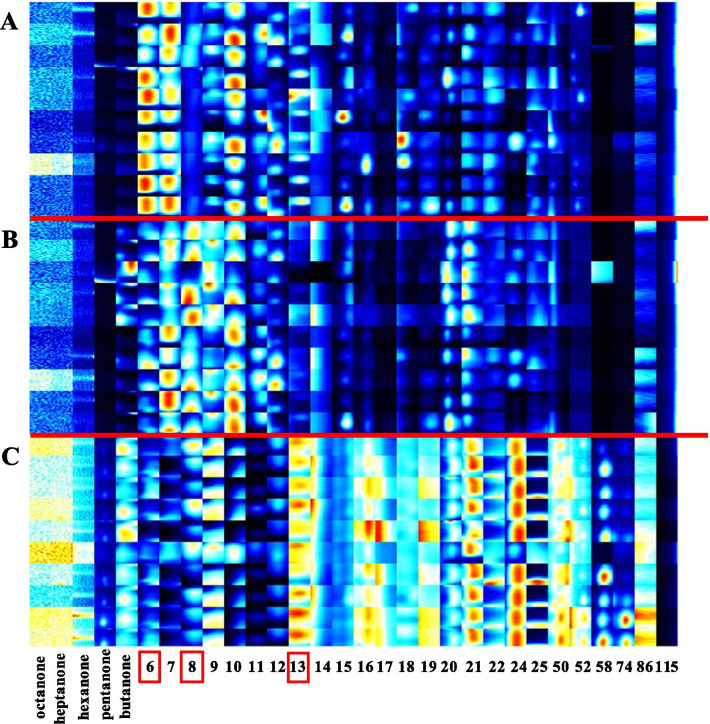
Comparison of preoperative OSCC patients with healthy controls. The heat map shows 25 areas of interest in 10 patients with OSCC (**a**), 10 healthy controls (**c**) and correlations with the room air of OSCC patients (**b**) in positive drift mode. Area 6 is significantly more pronounced in OSCC patients than room air, therefore, the endogenous origin of analytes can be assumed and may be associated with OSCC. In contrast, the signal observed in Area 8 is significantly increased in room air samples, an external origin of analytes is likely. The greatest signal intensity within Area 13 was observed for samples taken from the healthy control group

Signal intensity changes were also assessed. Many signals differed in intensity depending on whether the analysis was preoperative or postoperative, and an increased intensity was associated with an elevated concentration of the respective substance in a sample. In general, signals detected within postoperative samples tended to be elevated relative to preoperative samples. A comparison of OSCC patients and the control group showed that the number and intensity of signals in healthy participants was elevated relative to OSCC patients (Fig. 3). In some cases, the precise evaluation of the control group was difficult due to the presence of overlap between strongly pronounced signals.

### Machine learning

**Fig. 4:**
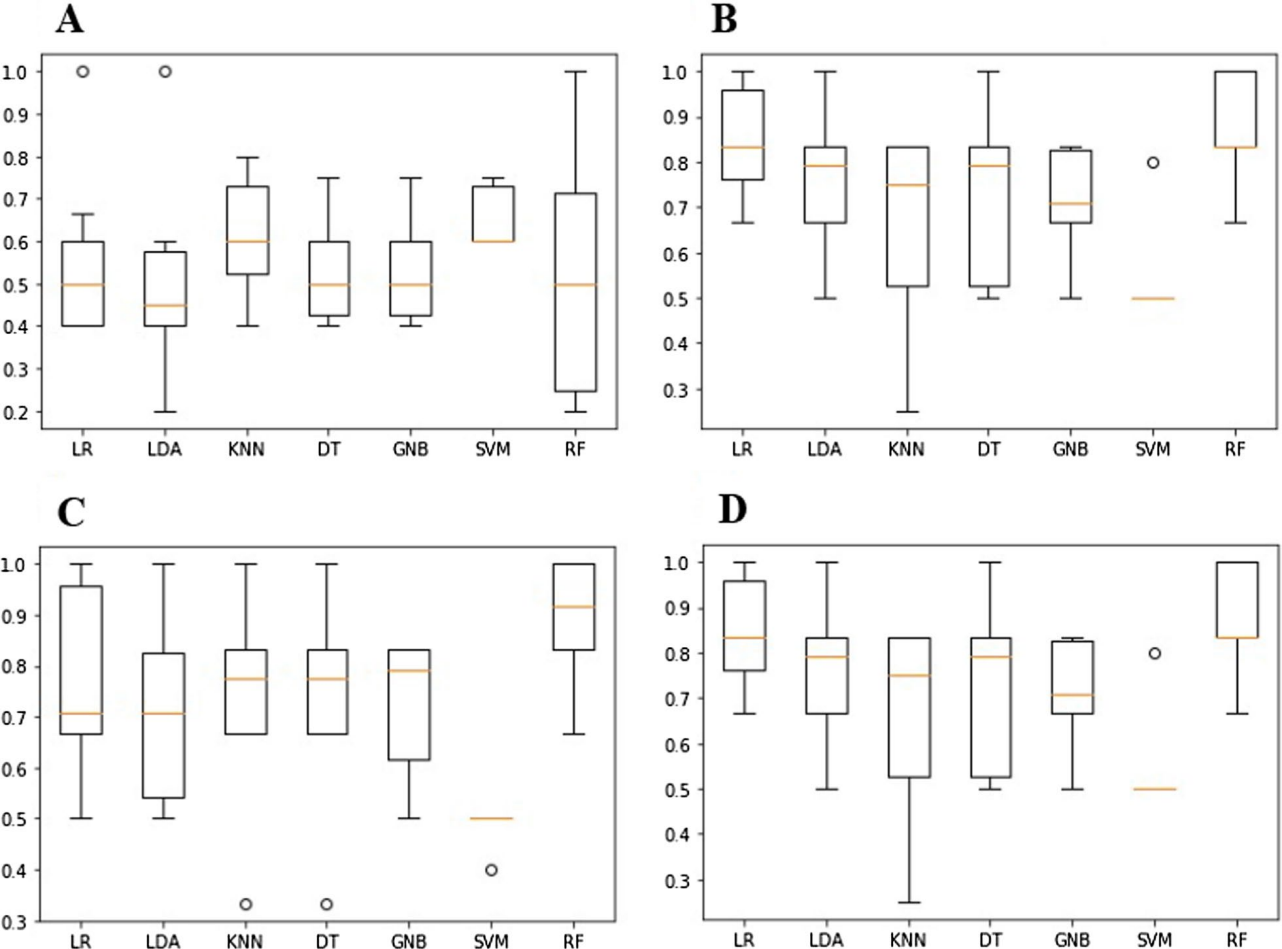
Comparison of the prediction accuracy of different machine learning models using tenfold cross-validation. **a** Pre- and post-operative samples in positive drift mode, **b** pre- and post-operative samples in negative drift mode, **c** pre-operative tumor samples and healthy smokers in positive drift mode, **d** pre-operative tumor samples and healthy smokers in negative drift mode are compared. *LR* logistic regression, *LDA* linear discriminant analysis, *KNN* k-nearest neighbors, *DT* decision tree, *GNB* gaussian naive bayes, *SVM* support vector machine, *RF* random forest

In the tenfold cross-validation process, pre- and postoperative samples in positive drift mode could only be distinguished with a highest average accuracy of 0.65 (Fig. 4a). For samples in negative drift mode, however, a highest average accuracy of 0.89 was obtained (Fig. 4b). Additionally, differentiating between preoperative tumor samples and healthy smoker samples using positive and negative drift mode could be done with a highest average accuracy of 0.90 and 0.86, respectively (Fig. 4c, d).

The estimated accuracy of the models was further confirmed using leave-one-out cross-validation, where logistic regression was determined to be the best performing method overall. For pre- and postoperative samples assessed in positive drift mode, 35 of 61 images (57%) were classified correctly (Additional file 1: Table S1). For samples assessed using negative drift mode this ratio improved to 43 of 58 (74%, Additional file 1: Table S2). Samples collected from preoperative tumor patients and healthy smokers were better differentiated. In samples assessed using positive drift mode, 60 of 72 samples (83%) were classified correctly (Additional file 1: Table S3), and in negative drift mode, 61 of 72 (85%) were predicted correctly (Additional file 1: Table S4). Additionally, we created sub-groups matching patients with either T1/2 (18 of 23) or T3/4 (28 of 33) tumors, female or male patients (16 of 35) and smoker or non-smoker (24/31) resulting in lower accuracies.

## Discussion and conclusion

This study showed that sampling exhaled air from the oral cavity using disposable syringes and subsequent processing is possible by following a standardized protocol. This eliminates the time-consuming intermediate step of storing samples before analyzing that has been used frequently to date [9, 38]. Sampling was a quick procedure that was easy to carry out and to learn for the practitioner. Since it is a non-invasive method, patient acceptance was very high. In this study, no patient refused to participate. Data analysis, however, was more complicated and required a trained user with current knowledge of the method. The targeted, pre-selection of relevant substances and automated analysis of specific patterns is needed to make breath testing user-friendly, error-free and widely applied in the future. A critical point in the study design was to define the healthy volunteers as smokers. The design aimed to make sure that signals from smoking habits would not mislead to the conclusion that by-products, from smoking are associated with OSCC, as also low-nicotine cigarettes lead to distortions in exhaled breath [39]. As some OSCC patients were self-reported non-smokers or former smokers, a control group should have been divided into smokers and non-smokers.

Various factors significantly influence the measurement data including food supply, oral hygiene, oral flora, the existence of other severe pre-existing malignant conditions, and the composition of air within the room [40–42]. To minimize these factors, samples from OSCC patients were consistently taken in the morning to observe a sobriety phase of at least 6 h. In addition, patients were asked to refrain from cleaning their teeth with toothpaste or mouthwash before sampling. Other pre-existing malignant conditions were an exclusion criterion for study participation. Even with these precautions, substances were present that were believed to be caused by food and oral hygiene products. A longer fasting episode may be necessary for completely eliminating these types of by-products. Two breath samples had to be stored in Mylar bags according to a widely accepted standard and we double-checked these samples prior to analysis, but ome compounds/signals may have been not stable until GC/IMS [31, 43]. It was difficult to ensure the sobriety of participants in the control group and prevent their use of oral hygiene products. This may have explained the enhanced intensity of signals observed for the group [44]. Also, substances from inhaled room air were recognizable in breath samples. Since these were hospital rooms, specific substances such as disinfectants were present in high quantities.

A comparison between OSCC patients and healthy smokers showed that certain substances were more prevalent in OSCC patients than healthy smokers (Fig. 3). For example, area 11 was significantly more pronounced in healthy participants than OSCC patients. Since area 11 was present in the lowest quantities in room air, it seems to be an endogenous human substance, which may be reduced as a result of OSCC. The structure of the compound should be evaluated in subsequent studies. A comparison between pre- and postoperative data revealed some substances that showed similar changes, e.g. the IMS signals of areas 4, 11, 13, 15, and 16 decreased postoperatively (Fig. 2, Table 3).

Our results showed that a detailed breakdown of single substances within samples is complex, and that patient compliance with detailed instructions is extremely important. The identification of purely endogenous substances associated with OSCC is difficult [45]. An increased intensity of signals in postoperative samples may be explained by worsened oral hygiene after surgery as a result of intraoral wounds [46].

Machine learning was able to distinguish between the OSCC patients and healthy volunteers. With an increased amount of data, the differentiation between pre- and postoperative patients might be possible as well to find out signals that may be emitted exclusively by tumor tissues. This is supported by the encouraging tenfold cross-validation result for samples in negative drift mode, where an average accuracy of 0.89 could be attained. This accuracy needs to be further evaluated with a larger patient cohort. In a larger cohort, a sub-group analysis of different tumor sizes, sex and smoking status will be interesting as well. Furthermore, it has to be noted, that the models are currently optimized to achieve an optimal overall accuracy.

At this stage, the testing of high-risk patients for OSCC is not yet feasible. Further studies focussing on (1) pattern recognition using machine learning in a larger cohort and (2) in vitro studies of tumor tissues using GC/MS to find out about specific VOCs with the help of libraries [47] must be carried out. The present study showed that breath sampling using GC/IMS was user-friendly and revealed results for the determination of OSCC in breath samples using machine learning with the highest achieved average accuracy of 86–90% when compared to healthy individuals. It also showed that breath sampling remains prone to interferences by by-products, so that further studies with much larger cohorts are necessary to remove interferences before going on with the development of an e-Nose that may be usable for early detection of OSCC.

## Supplementary Information

**Additional file 1**. Tables S1–4 provide details of the machine learning results.

## Data Availability

The datasets used are available from the corresponding author on reasonable request.

## Abbreviations

OSCC: Oral squamous cell carcinoma
GC–IMS: Gas chromatography coupled with ion mobility spectrometry
VOC: Volatile organic compounds
GC–MS: Gas chromatography coupled with mass spectrometry
ppb: Parts per billion
