# Supplementary material for: Prediction of oral squamous cell carcinoma based on machine learning of breath samples: a prospective controlled study
Source: BMC Oral Health. 2021 Oct 6;21:500. doi: 10.1186/s12903-021-01862-z (PMC8496028; doi:10.1186/s12903-021-01862-z)
Supplement: Supplementary file 1 — Additional file 1. Tables S1–4 provide details of the machine learning results. [file 12903_2021_1862_MOESM1_ESM.docx]

**Supplementary Material**

Supplemental Table 1: Results of leave-one-out cross-validation for pre- and post-operative samples in positive drift mode (61 images). LR: logistic regression, LDA: linear discriminant analysis, KNN: k-nearest neighbors, DT: decision tree, GNB: gaussian naive bayes, RF: random forest

|  | LR | LDA | KNN | DT | GNB | RF |
| --- | --- | --- | --- | --- | --- | --- |
| Correct predictions | 35 | 24 | 26 | 26 | 32 | 28 |
| False positives | 15 | 21 | 19 | 19 | 19 | 18 |
| False negatives | 11 | 16 | 16 | 16 | 10 | 15 |

Supplemental Table 2: Results of leave-one-out cross-validation for pre- and post-operative samples with negative ions (58 images). LR: logistic regression, LDA: linear discriminant analysis, KNN: k-nearest neighbors, DT: decision tree, GNB: gaussian naive bayes, RF: random forest

|  | LR | LDA | KNN | DT | GNB | RF |
| --- | --- | --- | --- | --- | --- | --- |
| Correct predictions | 43 | 37 | 31 | 41 | 38 | 34 |
| False positives | 10 | 12 | 16 | 8 | 12 | 13 |
| False negatives | 5 | 9 | 11 | 9 | 8 | 11 |

Supplemental Table 3: Results of leave-one-out cross-validation for pre-operative tumor samples and healthy smokers in positive drift mode (72 images). LR: logistic regression, LDA: linear discriminant analysis, KNN: k-nearest neighbors, DT: decision tree, GNB: gaussian naive bayes, RF: random forest

|  | LR | LDA | KNN | DT | GNB | RF |
| --- | --- | --- | --- | --- | --- | --- |
| Correct predictions | 60 | 60 | 52 | 64 | 50 | 62 |
| False positives | 4 | 4 | 6 | 3 | 18 | 3 |
| False negatives | 8 | 8 | 14 | 5 | 4 | 7 |

Supplemental Table 4: Results of leave-one-out cross-validation for pre-operative tumor samples and healthy smokers with negative ions (72 images). LR: logistic regression, LDA: linear discriminant analysis, KNN: k-nearest neighbors, DT: decision tree, GNB: gaussian naive bayes, RF: random forest

|  | LR | LDA | KNN | DT | GNB | RF |
| --- | --- | --- | --- | --- | --- | --- |
| Correct predictions | 61 | 58 | 49 | 49 | 52 | 59 |
| False positives | 5 | 10 | 7 | 14 | 6 | 4 |
| False negatives | 6 | 4 | 16 | 9 | 14 | 9 |
